# Wake up, College of Nursing

**Published:** 1918-12-28

**Authors:** 


### WHAT NURSES EXPECT AND WANT.

### Wake Up, College of Nursing.

The College of Nursing was founded and established as an Imperial Union of British Nurses with the object of ensuring betterment for its members, socially, economically, and professionally. This lofty ideal at once appealed to the neglected workers in a cause which should rank high in the economy of a progressive and civilising people, and so within a very short time ten thousand members had enrolled. Considering that there are quite seven times this number within the kingdom, the wonder is that this fine list is not greater. There are boundless possibilities to such a union as the College undertakes to form.

### A Reciprocal Duty to Fulfil.

It is apparent that the College Executive on the one hand, and its members on the other, have a reciprocal duty to fulfil, and it is doubtful if this is clearly and generally appreciated. The members joined in faith. They paid their £10,000 on the terms of the College prospectus, and there is scarcely room for doubt that they were inspired by the hope of deriving substantial and tangible benefit. Their object was not the building of an edifice which would be useful only to nurses of the next generation. I make this comment because it has been recently stated that economic benefit to the nurse will come by way of better education. I am not prepared either to affirm or to deny the truth of this statement, nor do I know whether it fully represents the official attitude of the College Executive. I hope it does not. To prescribe such a remedy for a serious complaint would very closely approximate to trifling. When the war broke out if England had informed France that no Expeditionary Force would be sent thither until a Channel Tunnel were constructed we should have lost the war, and, similarly, if the College elects to wait until by process of better education the nurse's economic conditions improve, it is highly probable that a scheme brilliantly conceived and successfully launched may end in failure.

### Constructive Criticism.

I have read and heard a variety of illfounded criticism of the College which merits no sympathy. It issues from a poisoned source, and is inspired by unworthy motives. My criticism is constructive, and what I desire to point out is that the response of the College Executive to its ten thousand members has so far been inadequate. Here and there hospital committees have raised the pay of their nurses. Here and there a trifle of consideration has been granted in more or less grudging fashion in respect of hours of labour and free time. But this cannot be accredited to any action taken by the College of Nursing. It is rather the result of the advocacy of that section of the nursing press which has championed the economic claims of the workers.

### What Ierne Expects.

What do I expect? I expect much, which I propose to set out. To begin with first principles, I hold the view that, founded on a democratic basis, the College union consists of its members, and that the bounden duty of the Executive is to discover their wishes and to endeavour to realise them. The business of a Member of Parliament is to voice his constituents. Otherwise the Bedford-Fenwick oft-repeated criticism that the College is a machine for "controlling" the profession will hold true. Hence the first and paramount duty of the College Executive is to put itself in touch with the individual members who make it what it is. It is not possible to gather these into the Albert Hall and hear them speak. But they can be consulted at their Centres or through the post. I, myself, think that I could make an intelligent guess as to their most pressing need. But, if I am cross-examined, I do not know, because I have not asked each individual member. Do the Executive of the College know? Are they any wiser than I in this respect? It is desirable that there should be some heart-searching. The one outstanding fact that presents itself to me is that there is not to-day in England any body of workers, save nurses, whose pay and conditions of service have been neglected. The day labourer in the city and on the land, the domestic servant, the woman clerk, the constable on the beat—all these have been borne on the tide of success to betterment, and they have been very largely helped forward by process of uniting. The nurse alone remains in statu quo ante bellum.

### "This is the Nurse's Hour."

The cry is "have patience, and it will all come right." I am afraid that my supply of this excellent virtue is limited. This is the nurse's hour, and many minutes have already passed. My ideals and those of the College are identical, and any difference that exists is only as regards procedure. The College is not, and never professed to be, a trades union. In no circumstances could countenance be given to a strike. Higher education, probity, honour, patriotism, devotion to duty, closer comradeship—of these the edifice is to consist. If it is so constructed the historian of a later generation will discern therein the metempsychosis of Florence Nightingale. But the building must start from an economic foundation, and it is with this that I am concerned. I have a lingering fear that the College is attempting the impossible task of building from the top. It is vain to talk to weary, underpaid, overworked women of "lectures," "examinations," "scholarships," and all the rest. At present she is a slave. Emancipate her, make her life tolerable, make it happy, and then begin the higher structure.

These are my views. Are they right or wrong? I claim to know, and I appeal to the rank and file to state in terms frank and free whether I am voicing their opinions, or, perchance, misrepresenting them. In like manner I appeal to the College. Let us have a full confession of faith. For more than a generation the British nurse has been pursuing shadows, with the result that she is still a bond slave, living on husks. Even at this late hour it is possible to strike out a path that leads otherwise than to the Kingdom of Nowhere. IERNE.

